# Antioxidant-independent activities of alpha-tocopherol

**DOI:** 10.1016/j.jbc.2025.108327

**Published:** 2025-02-18

**Authors:** Matthew Chen, Mikel Ghelfi, Jia-Fei Poon, Nayeon Jeon, Natalie Boccalon, Michael Rubsamen, Stephen Valentino, Vansh Mehta, Michaela Stamper, Hamza Tariq, Elizabeth Zunica, Lynn Ulatowski, Stacey Chung, Claire Fritz, Mark Cameron, Cheryl Cameron, Derek A. Pratt, Jeffrey Atkinson, Carrie J. Finno, Danny Manor

**Affiliations:** 1 Department of Nutrition, Case Western Reserve University, Cleveland, Ohio, USA; 2 Department of Chemistry, Brock University, Ontario, Canada; 3 Department of Chemistry and Biomolecular Sciences, University of Ottawa, Ottawa, Ontario, Canada; 4 Department of Biology, Ursuline College, Pepper Pike, Ohio, USA; 5 Department of Population Health and Reproduction, School of Veterinary Medicine, University of California Davis, Davis, California, USA

**Keywords:** tocopherol, vitamin E, gene expression, lipids, vitamins

## Abstract

Alpha-tocopherol (vitamin E) is a plant-derived dietary lipid that is essential for the health of most animals, including humans. Originally discovered as a fertility factor in rodents, the primary health-promoting properties of the vitamin in humans was shown to be protection of neuromuscular functions. Heritable vitamin E deficiency manifests in spinocerebellar ataxia that can be stabilized by timely supplementation with high-dose α-tocopherol. The molecular basis for α-tocopherol's biological activities has been attributed primarily to the vitamin's efficacy in preventing lipid peroxidation in membranes and lipoproteins, but the possibility that the vitamin possesses additional biological activities has been postulated and debated in the literature without conclusive resolution. We designed and synthesized a novel analog of α-tocopherol, 6-hydroxymethyl α-tocopherol (6-HMTC), which retains most of the vitamin's structural, physical, and biochemical properties, yet lacks measurable radical-trapping antioxidant activity. 6-HMTC bound to the tocopherol transfer protein with high (nanomolar) affinity, like that of the natural vitamin, attesting to the analog's preservation of structural integrity. Yet, 6-HMTC did not inhibit lipid peroxidation or associated ferroptotic cell death. Notably, 6-HMTC modulated the expression of some genes in a manner essentially identical to that exhibited by α-tocopherol. These findings support the notion that α-tocopherol modulates gene expression *via* an antioxidant-independent mechanism.

The term vitamin E refers to a family of neutral plant-derived lipids, of which α-tocopherol is selectively retained in tissues of most animals and is considered the most biologically active form of the vitamin (1, 2, 3). The vitamin's relevance to health was initially recognized as a fertility-promoting factor in rodents (4) and later as a neuroprotective agent in humans and other animals (5, 6, 7, 8). The mechanistic basis for α-tocopherol's health-promoting actions has been ascribed to the molecule's potent radical-trapping antioxidant activity, which prevents free radical–mediated lipid peroxidation. Thus, the vitamin is considered to be the major lipid-soluble antioxidant in most species (9, 10). Consequently, adequate vitamin E status is thought to be an important line of physiological defense against some oxidative stress–related pathologies (11, 12), particularly those wherein ferroptosis—cell death associated with unrestrained lipid peroxidation—has been implicated.

The notion that vitamin E possesses additional biological activities that are not related to the molecule's established radical-trapping activity has been repeatedly discussed in the literature. Of special interest are multiple studies that report alterations in gene expression patterns under different vitamin E levels (13, 14, 15, 16, 17, 18). These observations were further supported by studies that reported changes in gene expression profiles that are unique to α-tocopherol when compared with other antioxidants (19, 20) and by the known transcriptional activities of other fat-soluble micronutrients, such as vitamins A, D, and K. However, conclusive proof that vitamin E has antioxidant-independent action is complicated by the global transcriptomic to changes in the cellular redox status and antioxidants, such as those mediated the transcription factor Nrf2 (21).

To conclusively examine the possibility of antioxidant-independent transcriptional activities of vitamin E, we have synthesized and characterized the redox-inert analog of vitamin E, 6-hydroxymethyl α-tocopherol (6-HMTC) and investigated its functionality in modulating gene expression in cultured cells.

## Results and discussion

The absolute requirement of α-tocopherol for optimal health has been established over a hundred years ago (4). Numerous studies demonstrated that α-tocopherol is an essential nutrient that is highly effective as a lipid-soluble antioxidant, that is, it reacts quickly with peroxyl radicals formed within biological membranes *in vitro* as well as *in vivo*, thereby preventing propagation of the lipid peroxidation radical chain reaction (1, 2, 9, 22). Moreover, it has been established that the vitamin's radical-trapping antioxidant activity underlies at least some of its biological actions (23). Nevertheless, reports of biological activities of vitamin E that are distinct from, and independent of, its antioxidant activity have also been documented in the literature. Such claims have primarily been based on biological activities of α-tocopherol that are not shared by structurally similar antioxidants, such as other members of the vitamin E family, or by other antioxidants (*cf.* (24)). In addition, claims of exclusive antioxidant mechanisms of action were not supported by clinical trials where vitamin E supplementation failed to show conclusive benefits in disease states that are thought to be mediated by oxidative stress (*e.g.*, (25, 26, 27, 28)). These reports gave rise to the notion that α-tocopherol may possess novel activities that are independent of its capacity to inhibit lipid peroxidation and generated a lively debate in the literature (24, 29, 30). Considering the critical importance of vitamin E in human health, we sought to conclusively address this question by examining the biological activities of a vitamin E analog that retains most of α-tocopherol's physicochemical properties yet is inert as an antioxidant.

### Design and synthesis of 6-hydroxymethyl-*RRR*-α-tocopherol (6-HMTC)

**Figure 1:**
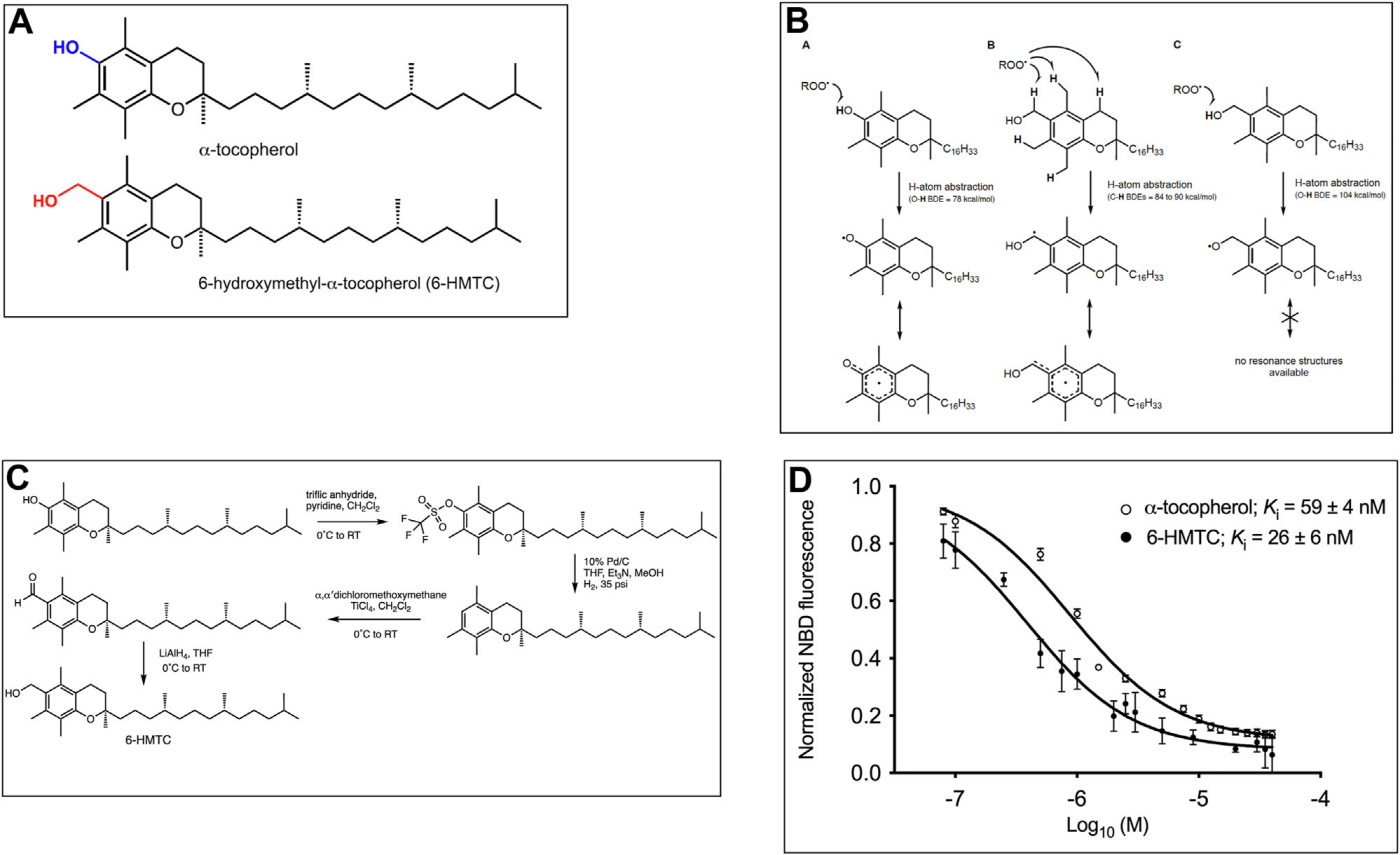
**6-HMTC is an analog of vitamin E with no antioxidant activity *in vitro*.***A*, chemical structures of α-tocopherol (*top*) and of 6-HMTC (*bottom*). *B*, rationale for the poor radical-trapping activity of 6-HMTC. Shown are possible hydrogen-atom abstractions by lipid peroxyl radicals from benzylic positions of (*A*) alpha-tocopherol, (*B*) 6-HMTC, and (*C*), from the benzylic alcohol of 6-HMTC. *A*, the established mechanism of tocopherol acting as a chain-terminating antioxidant. *B*, the possible sites of benzylic H-atom abstraction from 6-HMTC. *C*, depicts how the alcohol of 6-HMTC has the highest BDE. *C*, schematic scheme for the synthesis of 6-HMTC. See Supporting Information for details. *D*, high-affinity binding of 6-HMTC to the α-tocopherol transfer protein (TTP). Purified recombinant TTP (0.4 μM) was preincubated with 1.6 μM NBD-C9-α-Toc in 250 mM sucrose, 100 mM KCl, 50 mM Tris, 1 mM EDTA, pH 7.4. Incremental amounts of unlabeled α-tocopherol (*empty circles*) or 6-HMTC (*solid circles*) were added, and fluorescence values recorded for each addition after equilibrium were reached (15 min). BDE, bond dissociation enthalpy; 6-HMTC, 6-hydroxymethyl α-tocopherol.

To investigate whether the biological actions of α-tocopherol involve antioxidant-independent mechanisms, we designed a synthetic analog that has no radical-trapping activity, while retaining all other relevant molecular features of α-tocopherol. To this end, we synthesized 6-hydroxymethyl α-tocopherol (6-HMTC), in which the vitamin's phenolic hydroxyl group is replaced with a hydroxymethyl group (Fig. 1*A*). The phenolic O-H moiety of α-tocopherol has a low bond dissociation enthalpy of ∼78 kcal/mol (31), whereas that of the hydroxymethyl O-H group in 6-HMTC is expected to be that of a typical aliphatic alcohol (∼104 kcal/mol (32, 33)), such that if H-atom transfer were to occur, it would be from a benzylic C-H bond instead (bond dissociation enthalpy: 84–90 kcal/mol (33); Fig. 1*B*). Since H-atom transfer from a benzylic C-H bond to a peroxyl radical is comparatively slow (*cf. k* = 0.34 for cumene compared with *k* = 3 × 10^6^ M^−1^s^−1^ for α-tocopherol; (34)), Therefore, 6-HMTC is expected to be inactive as a radical-trapping antioxidant.

The synthetic scheme (described in Fig. 1*C* and in the Supporting Information) has allowed for the preparation of gram amounts of 6-HMTC with purity of >98%. To evaluate the analog's structural similarity to α-tocopherol, we measured its binding affinity to the tocopherol transfer protein (TTP, (6, 35, 36, 37, 38, 39)). The protein accommodates vitamin E within a hydrophobic binding pocket (40, 41), to which α-tocopherol binds with nanomolar affinity and high degree of ligand discrimination (42, 43). To quantify the affinity of 6-HMTC for TTP, we measured its ability to displace a fluorescent derivative of α-tocopherol from the protein's binding pocket using a fluorescence competition assay we characterized extensively (44, 45, 46, 47, 48). We found that 6-HMTC binds to TTP reversibly and with high (nanomolar) affinity, very similar to that displayed by the native vitamin (Fig. 1*D*).

**Figure 2:**
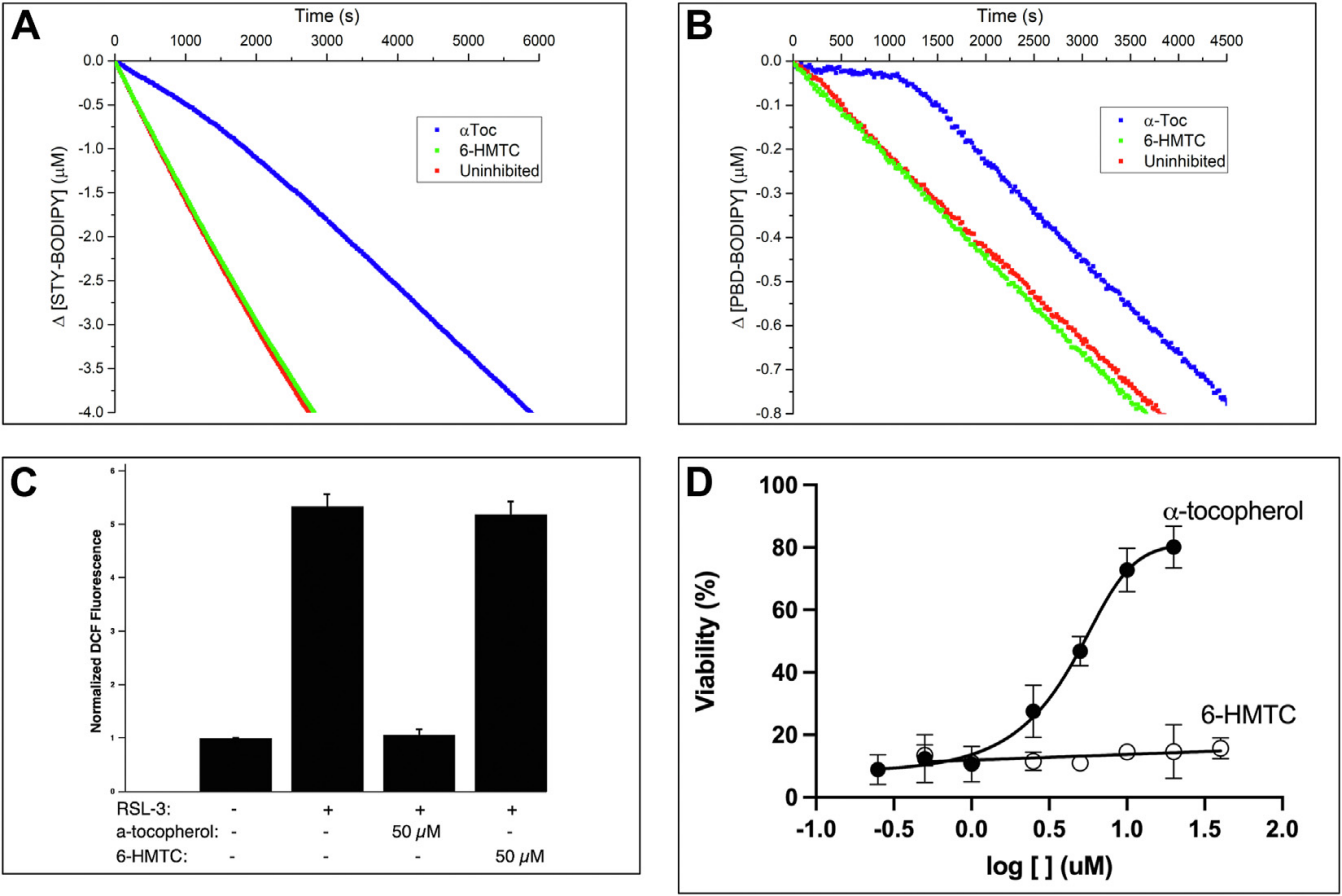
**6-HMTC has no antioxidant activity *in vitro* and *in vivo*.***A*, coautoxidation of egg phosphatidylcholine lipids (1 mM) and STY-BODIPY (8 μM) suspended in phosphate-buffered saline, pH 7.4 was initiated by di-*tert*-undecylhyponitrite (DTUN; 0.2 mM) at 37 °C. Oxidation was inhibited by the presence of 10 μM α-tocopherol (*blue*), whereas 6-HMTC (*red*) did not impact lipid oxidation under the same conditions. *B*, coautoxidation of styrene (4.3 M) and PBD-BODIPY (10 μM) was initiated by AIBN (6 mM) in phenyl chloride at 37°C and was delayed in the presence of 2 μM α-tocopherol (*blue*). 6-HMTC (*red*) was ineffective in delaying lipid oxidation under the same conditions. *C*, accumulation of lipid hydroxides was induced in cultured IHH cells by treatment with RSL-3, and intracellular reactive oxygen species were measured using the cell-permeable fluorescent probe CM-H2DCFDA (2′,7′-dichlorofluorescin diacetate; 20 μM). Where indicated, cells were preincubated with 50 μM α-tocopherol, 6-HMTC, or control solvent (*p < 0.01*). *D*, 6-HMTC does not protect cells from RSL-3-induced ferroptosis. Human embryonic kidney 293 cells were pretreated with the indicated concentrations (0, 0.25, 0.5, 1, 2.5, 5, 10, 20, and 40 μM) of α-tocopherol (*solid circles*) or 6-HMTC (*empty circles*) prior to incubation with RSL-3 (0.45 μM; 3.5 h). Viability was measured using the AquaBluer assay (MultiTarget Pharmaceuticals) according to the manufacturer's instructions. Cell viability was calculated by normalizing the data to untreated controls. Each experiment was carried out in six analytical replicates per concentration and repeated independently three times. 6-HMTC, 6-hydroxymethyl α-tocopherol; AIBN, azobisisobutyronitrile; IHH, immortalized human hepatocyte; RSL-3, Ras-selective lethal 3.

Next, we tested the ability of 6-HMTC to prevent lipid peroxidation *in vitro*. As expected, α-tocopherol efficiently inhibited the peroxidation of membrane phospholipids as judged by its effect on the autoxidation of unilammelar liposomes of egg phosphatidylcholine induced by the lipophilic hyponitrite DTUN (di-*tert*-undecylhyponitrite, (49); Fig. 2*A*). Under identical conditions, 6-HMTC did not inhibit lipid oxidation. From the initial rates of oxidation of the fluorescent reporter STY-BODIPY, the rate constant for reaction with chain-carrying lipid peroxyl radicals is 1.8 × 10^3^ M^−1^s^−1^, in agreement with previously reported values (50). The lack of inhibition by 6-HTMC implies that its reactivity is <<10^3^ M^−1^s^−1^, such that it cannot effectively compete with chain propagation. Since the intrinsic reactivity of radical-trapping antioxidants with peroxyl radicals is more easily revealed in inhibited autoxidation of simple hydrocarbons, we also carried out azobis(isobutyronitrile)–initiated autoxidation of styrene (51)). Again, while α-tocopherol efficiently inhibited peroxidation with a rate constant fully consistent with literature values (1.9 × 10^6^ M^−1^s^−1^), 6-HTMC did not show any inhibitory activity (Fig. 2*B*).

Next, we compared the ability of α-tocopherol and 6-HMTC to inhibit lipid peroxidation in live cells. We treated immortalized human hepatocytes (IHHs, (52, 53)) with RSL-3 (Ras-selective lethal 3), an inhibitor of glutathione peroxidase 4 (GPX4; (51)). Since GPX4's enzymatic activity eliminates phospholipid hydroperoxides (54, 55), its inhibition with RSL-3 leads to marked increase in the levels of intracellular reactive oxygen species, as reported previously (55) and as shown in Figure 2, using the oxidation-sensitive fluorescent probe dichlorofluorescin (DCF) diacetate. Pretreatment of the cells with vitamin E (50 μM α-tocopherol) completely abolished the RSL-3-induced increase in DCF fluorescence, in accordance with the vitamin's established antioxidant activity (Fig. 2*C*). 6-HMTC, on the other hand, was completely ineffective in attenuating the RSL-3-induced increase in DCF fluorescence, indicating that the analog has no measurable antioxidant activity in cells.

Further support for this notion was obtained when cell viability was assessed following prolonged GPX4 inhibition, which induces ferroptotic cell death (54, 56, 57). As expected, pretreatment with α-tocopherol protected cells from the toxic effect of RSL-3, whereas 6-HMTC did not impact the cells' sensitivity to GPX4 inhibition (Fig. 2*D*). Taken together, these data show that our design and synthesis of 6-HMTC produced an analog of α-tocopherol that resembles the native vitamin structurally, yet has no detectable antioxidant radical trapping activity. Thus, 6-HMTC is suitable for dissecting the biological roles of vitamin E's antioxidant function from those that are independent of this activity.

**Figure 3:**
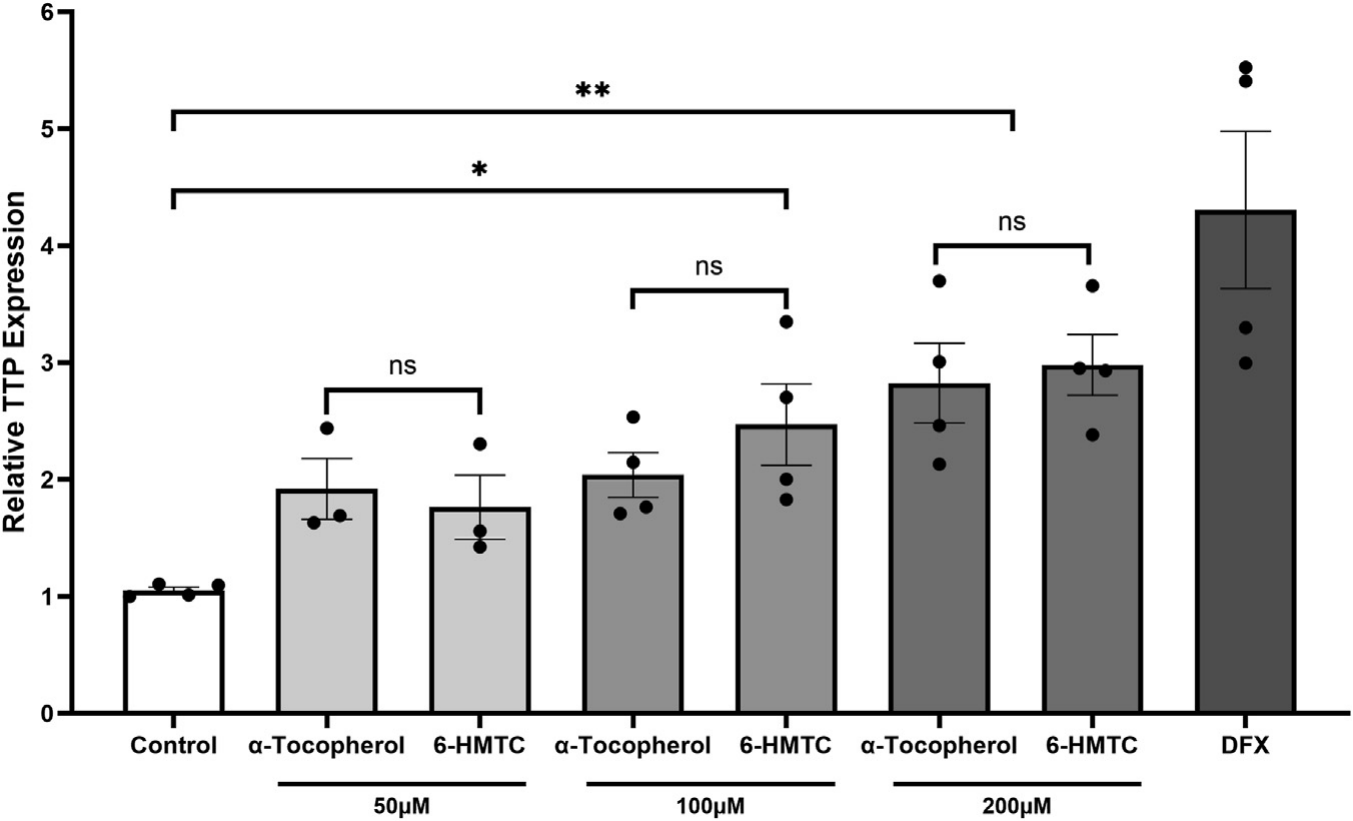
**Transcription of the *TTPA* gene is enhanced by α-tocopherol as well as 6-HMTC.** Triplicate IHH cultures were treated with the indicated compound for 24 h prior to RT–PCR analyses of the *TTPA* transcript as described in Experimental procedures section. Transcript abundance was normalized to that of a housekeeping transcript (GAPDH) and then normalized to the value obtained for untreated cells. DFX (deferoxamine) is a hypoxia-inducing iron chelator that was previously shown to increase *TTPA* levels (62). Shown are means ± standard deviations of the data. 6-HMTC, 6-hydroxymethyl α-tocopherol; IHH, immortalized human hepatocyte.

Multiple reports documented a profound impact of vitamin E on gene expression, in cultured cells as well as animals (*cf.* (24, 58)). Some of these studies compared gene expression profiles between vitamin E–deficient and vitamin E–sufficient physiological states (*e.g.*, (13, 59, 60)). Interpretation of these results is challenging in light of the profound global impact of oxidative stress on gene expression, mediated by redox-sensitive transcription factors (*e.g.*, Nrf2, (21)). Other studies compared the impact of α-tocopherol on gene expression to that of other members of the vitamin E family (*e.g.*, γ-tocopherol, (17)) or to other antioxidants (*e.g.*, *N*-acetyl-cysteine, (19)), but observed differences could stem from altered bioavailability, routes of uptake, or metabolic fate of the different molecules. Thus, it is not surprising that a mechanistic model explaining *how* vitamin E regulates gene expression is yet to be presented. The advent of a redox-inert analog of α-tocopherol allowed us to address this enigma directly, by comparing the transcriptomic profile of cells treated with vitamin E *versus* those treated with 6-HMTC. We treated immortalized human hepatocytes with either α-tocopherol or 6-HMTC and used real-time RT–PCR to quantify the levels of the mRNA transcripts encoding the TTP (36, 61), which were previously shown to increase upon vitamin E treatment of these cells (62). As shown in Fig. 3, both α-tocopherol and 6-HMTC increased *TTPA* expression in a manner indistinguishable from each other (*p* < 0.05). These data indicate that vitamin E–induced increase in *TTPA* mRNA levels occurs through an antioxidant-independent mechanism.

**Figure 4:**
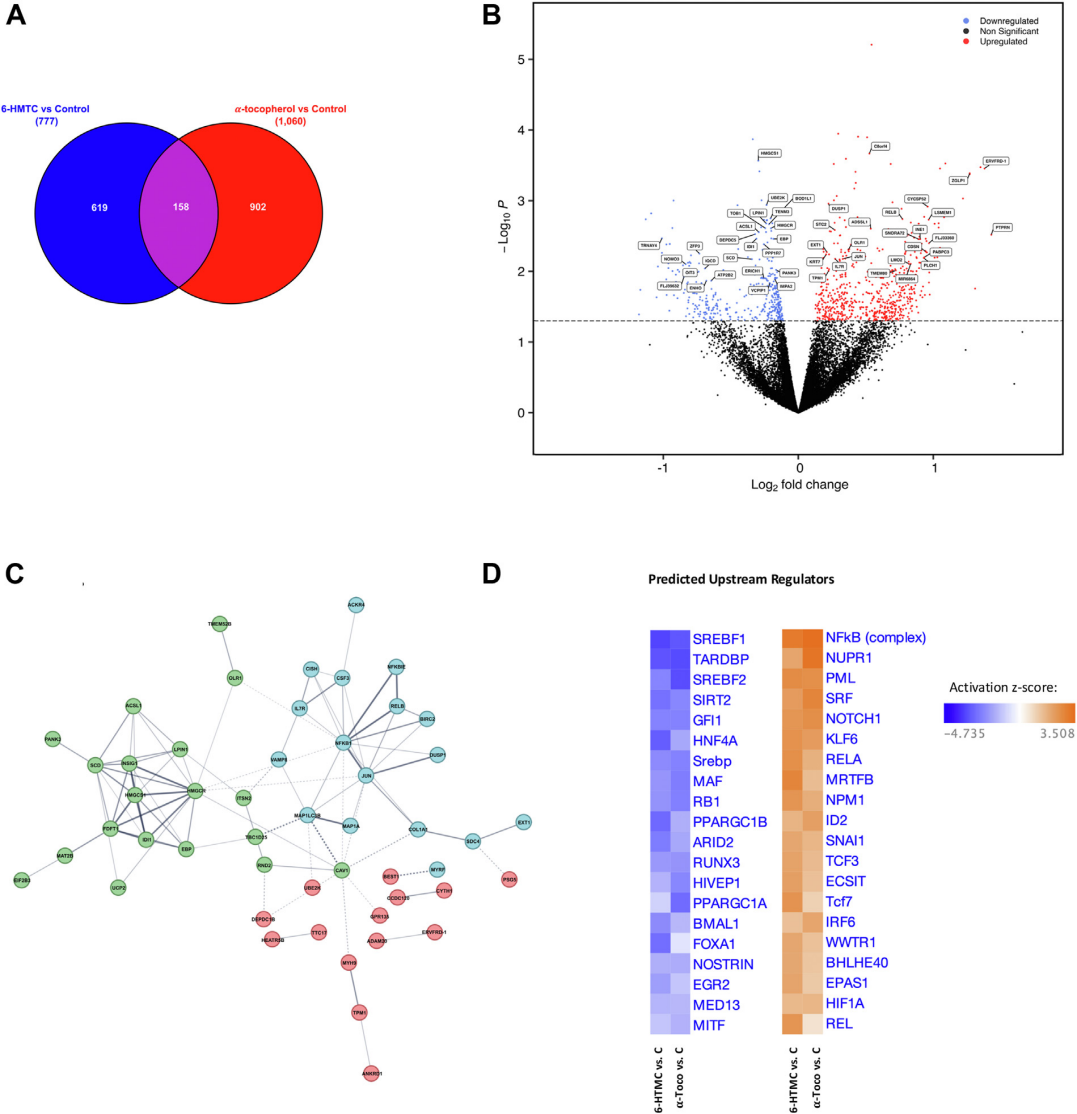
**Differential gene expression profiles of vitamin E- and 6-HMTC-treated IHH cells.** IHH cells were treated with 50 μM α-tocopherol, 50 μM 6-HMTC, or dimethyl sulfoxide control for 24 h and subjected to RNA-Seq. *A*, Venn diagram summarizing transcriptome changes in each treatment group. *B*, volcano plot illustrating the statistical significance and fold change of each gene in the a-tocopherol *versus* control contrast. The top 25 upregulated and top 25 downregulated genes that were differentially expressed across both the 6-HMTC *versus* control and the α-tocopherol *versus* control groups are labeled. *C*, protein–protein interaction (PPI) network illustrating the relationship between all 158 statistically significant differentially expressed genes shared amongst the 6-HMTC *versus* control and a-tocopherol *versus* control contrasts. K-means clustering was used to define discrete clusters, each colored in different colors. *D*, heatmap showing the top predicted transcription factors that could mediate the changes in transcript levels observed within each contrast. 6-HMTC, 6-hydroxymethyl α-tocopherol; IHH, immortalized human hepatocyte.

These findings prompted us to undertake an unbiased transcriptomic approach aimed at deciphering the global transcriptional responses to α-tocopherol and 6-HMTC, respectively. Using a similar experimental design, we employed RNA-Seq to profile the transcriptome response to each of the two molecules. We found that the levels of 158 mRNA transcripts were impacted similarly by α-tocopherol and 6-HMTC as compared with control-treated cells (84 upregulated and 74 downregulated mRNAs; *p*_unadjusted_ ≤0.05; Fig. 4, *A* and *B*, Tables 1 and 2, and S1).

**Table 1:** Top genes upregulated by both HTMC and α-tocopherol

Gene symbol	Assigned gene name	Assigned function
C8orf4	Transcriptional And Immune Response Regulator	A positive regulator of the Wnt/beta-catenin signaling pathway. This protein interacts with a repressor of beta-catenin-mediated transcription at nuclear speckles
ZGLP1	Zinc Finger GATA Like Protein 1	Transcriptional regulator with GATA-like zinc fingers
DUSP1	Dual Specificity Phosphatase 1	Tyrosine and Ser/Thr phosphatase that can deactivate MAPK1/ERK2. Important in the cellular response to environmental stress
RELB	RELB Proto-Oncogene, NF-KB Subunit	A member of the NFKB family of transcription factors that functions in inflammatory responses
LSMEM1	Leucine Rich Single-Pass Membrane Protein 1	Conserved protein of unknown function
ADSSL1	Adenylosuccinate Synthase 1	A muscle-specific enzyme that catalyzes the first step in the conversion of inosine monophosphate (IMP) to AMP
STC2	Stanniocalcin 2	Stress-induced protein that potentially mediates the response to nutrient sufficiency
PTPRN	Protein Tyrosine Phosphatase Receptor Type N	A tyrosine phosphatase that regulates the vesicular secretion of some hormones
INE1	Inactivation Escape 1	X-linked lncRNA
SNORA72	Small Nucleolar RNA	Noncoding small RNA located in the nucleolus
FLJ33360	Long Intergenic Non-Protein Coding RNA 2145	lncRNA thought to function in the sequestration of miRNAs
OLR1	Oxidized Low Density Lipoprotein Receptor 1	A receptor of the lectin superfamily that degrades oxidized low-density lipoproteins
CDSN	Corneodesmosin	A protein found in corneodesmosomes and functions in skin integrity
EXT1	Exostosin Glycosyltransferase 1	An endoplasmic reticulum–resident glycosyltransferase involved in heparan sulfate biosynthesis
KRT7	Keratin 7	A type II cytokeratin expressed in simple and stratified epithelial tissues
PABPC3	Poly(A) Binding Protein Cytoplasmic 3	Regulates mRNA stability and translation initiation
PLCH1	Phospholipase C Eta 1	A phosphoinositide-specific phospholipase C that cleave phosphatidylinositol 4,5-bisphosphate to inositol 1,4,5-trisphosphate (IP3) and diacylglycerol (DAG)
IL7R	Interleukin 7 Receptor	Receptor for interleukin 7 that functions primarily in T-cell biology
JUN	Jun Proto-Oncogene	A functional component of the AP1 transcription factor complex
MIR6864	MicroRNA 6864	A noncoding RNA involved in post-transcriptional regulation of gene expression
LMO2	LIM Domain Only 2	A cysteine-rich transcription factor that functions in hematopoiesis
TMEM80	Transmembrane Protein 80	Nonmotile cilium-associated protein
TPM1	Tropomyosin 1	Actin-binding protein involved in the contraction of muscles and the cytoskeleton of nonmuscle cells

**Table 2:** Top genes downregulated by both HTMC and α-tocopherol

Gene Symbol	Assigned gene name	Assigned function
HMGCS1	3-Hydroxy-3-Methylglutaryl-CoA Synthase 1	Catalyzes the conversion of acetoacetyl-CoA into 3-hydroxy-3-methylglutaryl-CoA (HMG-CoA) in the cholesterol biosynthesis pathway
UBE2K	Ubiquitin Conjugating Enzyme E2 K	An E2 ubiquitin-conjugating enzymes that mediates proteasomal targeting and degradation of other proteins
TENM3	Teneurin Transmembrane Protein 3	Regulates connectivity within the nervous system
LPIN1	Lipin 1	A phosphatidic acid phosphohydrolase that catalyzes the penultimate step in triglyceride synthesis
BOD1L1	Biorientation Of Chromosomes In Cell Division 1 Like 1	Component of the replication fork
TOB1	Transducer Of ERBB2, 1	Anti-proliferative factor that controls cell cycle progression
HMGCR	3-Hydroxy-3-Methylglutaryl-CoA Reductase	Catalyzes the rate-limiting enzyme for cholesterol synthesis. Target of statin-family of cholesterol-lowering drugs
ACSL1	Acyl-CoA Synthetase Long Chain Family Member 1	A long-chain fatty-acid-coenzyme A ligase
DEPDC5	DEP Domain Containing 5, GATOR1 Subcomplex Subunit	Component of the GATOR1 complex that regulates mTor signaling
IDI1	Isopentenyl-Diphosphate Delta Isomerase 1	Catalyzes the interconversion of isopentenyl diphosphate (IPP) to dimethylallyl diphosphate (DMAPP), in *de novo* biosynthesis of cholesterol
EBP	Emopamil binding protein; 3β-hydroxysteroid-Δ8,Δ7-isomerase	Converts 8(9)-cholestenol to lathosterol in the pathway of *de novo* cholesterol biosynthesis
PPP1R7	Protein Phosphatase 1 Regulatory Subunit 7	Regulatory subunit of the serine/threonine phosphatase, protein phosphatase-1
ZFP3	ZFP3 Zinc Finger Protein	Predicted DNA-binding transcription factor
SCD	Stearoyl-CoA Desaturase	Catalyzes a rate-limiting step in the synthesis of unsaturated fatty acids, converting stearic acid to oleic acid
NOMO3	NODAL Modulator 3	Participates in the Nodal signaling pathway during vertebrate development
IQCD	IQ Motif Containing D	Component of the nexin-dynein regulatory complex that regulates cytoskeleton and cilia motility
PANK3	Pantothenate Kinase 3	Catalyzes the phosphorylation of pantothenate to generate 4′-phosphopantothenate in the first and rate-determining step of coenzyme A (CoA) synthesis
OIT3	Oncoprotein Induced Transcript 3; LZP	Liver-expressed protein involved in lipid metabolism and carcinogenesis
ERICH1	Glutamate Rich 1	Protein of unknown function
IMPA2	Inositol Monophosphatase 2	Dephosphoylrates inositol monophosphate and plays an important role in phosphatidylinositol signaling
ENHO	Energy Homeostasis-Associated Protein; Adropin	Secreted peptide hormone involved in hepatic carbohydrate and lipoprotein metabolism
ATP2B2	ATPase Plasma Membrane Ca2+ Transporting 2	ATP-driven Ca^2+^ ion transporter
VCPIP1	Valosin Containing Protein Interacting Protein 1	Thiol-dependent protein deubiquitinase involved in DNA repair and Golgi and endoplasmic reticulum biogenesis

The top downregulated transcript shared between the α-tocopherol and 6-HMTC treatment groups (Fig. 4, *B* and *C*, Tables 1, and S1) included *HMGCS1* (*3-hydroxy-3-methylglutaryl-CoA synthase 1*), the first enzyme in the *de novo* biosynthesis of cholesterol, which catalyzes the conversion of acetyl-CoA (coenzyme A) to HMG-CoA. Interestingly, additional cholesterol metabolism genes were downregulated by both treatments, including (1) *HMGCR* (*3-hydroxy-3-methylglutaryl-CoA reductase*), the rate-limiting enzyme of the pathway and target of statins; (2) *IDI1* (*isopentenyl-diphosphate delta isomerase 1*), that catalyzes the synthesis of farnesyl diphosphate; (3) *EPB* (*emopamil binding protein*), a sterol isomerase; and (4) FDFD1 (*squalene synthase*), both of which participate in the final steps of cholesterol biosynthesis; (5) *SCD* (*stearoyl-CoA desaturase*) catalyzes the rate-limiting step in the formation of monounsaturated fatty acids, which are then used to synthesize cholesterol esters; (6) *PANK3* (*pantothenate kinase 3*), the first enzyme in the CoA biosynthetic pathway; and (7) the endoplasmic reticulum sterol sensor INSIG1. Top transcripts that were similarly increased by both treatments included components of the NFKB signaling system, namely NFKB1, NFKBIE, and RELB, in addition to the transcriptional regulator *JUN*, the inhibitor of apoptosis *BIRC2*, and the dual-specificity phosphatase DUSP1 (Tables 2 and S1).

We evaluated the likely physiological consequences of vitamin E's transcriptional activities, using STRING (63), a database of known and predicted protein–protein interactions, to compare the transcriptomic changes to α-tocopherol *versus* control with those of 6-HTMC *versus* control. In accordance with the individual transcript changes listed previously, this analysis indeed predicted that vitamin E treatment causes a *functional* inhibition of cholesterol synthesis as well as stimulation of NF-kB-mediated immune responses (Fig. 4*C*).

The attenuation of cholesterol biosynthesis by vitamin E may be of profound translational and clinical significance. Notably, multiple reports documented a cholesterol-lowering effect of vitamin E treatments both *in vivo* and *in vitro* (64, 65, 66, 67, 68, 69). Moreover, experiments in cultured HepG2 cells showed that vitamin E attenuates the metabolic flux through this pathway (19). Such a cholesterol-lowering effect could lower human risk for cardiovascular disease, as was thought during the 1990s (70, 71), but conflicting data and concerns of unintended side effects hampered enthusiasm at the time. Our results, together with recent studies demonstrating the safety of vitamin E supplementation (72, 73), may open the door for re-evaluation of vitamin E's cardioprotective actions. Importantly, our findings indicate that the interplay between tocopherol status and cholesterol levels possibly originates from a novel antioxidant-independent function of the vitamin. The molecular mechanisms that underlie this phenomenon are presently unknown, but it is tempting to consider the possibility that established pathway of cholesterol homeostasis, in which sterol levels are sensed by INSIG and impact the transcriptional activities of sterol regulatory element–binding protein (SREBP) *via* its regulator SREBP cleavage-activating protein (74), plays a role in mediating vitamin E's actions. Future work will address whether vitamin E directly binds to INSIG1, thereby impacting this established pathway (75).

Another important transcriptional effect shared by both 6-HMTC and α-tocopherol treatments is the modulation of immune processes and inflammatory responses, evidenced by the transcriptional upregulation of the inflammatory regulators NFKB1, NFKBIE, DUSP1, and RELB (Table 2 and Fig. 4*C*, *blue circles*). Although previous reports demonstrated anti-inflammatory actions of vitamin E (76) and vitamin E–induced increases in transcripts of the NF-κB pathway (77), we did not observe changes in transcripts of its classical downstream targets such as tumor necrosis factor alpha or interleukin-1 (78). This, perhaps is not surprising since these mediators typically act in cells of the immune system, rather than in terminally differentiated cells such as the hepatocytes in our study. Future studies will address the possibility that vitamin E–induced Nf-κB activation in hepatocytes leads to noninflammatory outcomes of the pathway, such as its reported antiapoptotic signaling (79), responses to redox changes (80) and/or injury responses (81, 82).

A critical unanswered question involves the molecular mechanisms by which vitamin E impacts the transcriptome. We mined the dataset using the ingenuity pathway analysis (Qiagen, Inc) for common transcription factors that might be responsible for changes in these transcripts, shown in Figure 4*D*. This analysis confirmed the importance of the transcriptional regulators discussed previously, that is, SREBP cleavage–activating protein and NF-kB, as well as multiple general transcriptional regulators (*e.g.*, RUNX3, FOXA1, SRF, KLF6).

In addition, ingenuity pathway analysis identified the nuclear receptor peroxisome proliferator–activated receptor gamma (PPARγ) as a possible mediator of vitamin E's transcriptional responses. Since the activity of most nuclear receptors is regulated by small tightly bound hydrophobic ligands, and since they mediate the genomic activities of other fat-soluble vitamins and their metabolites (83), we sought to test whether α-tocopherol *directly* activates specific nuclear receptors. Toward this end, we used established *in vitro* transactivation reporter assays that examine the ability of a small molecule to modulate the transcriptional activity of a nuclear receptor that drives expression of a reporter gene (84). Specifically, we tested whether α-tocopherol alters the activity of the three peroxisome proliferator–activator receptors (PPARα, PPARβ, or PPARγ), the liver X receptor, the pregnane X receptor, or the farnesoid X receptor. The data indicate that α-tocopherol (50–100 μM from either ethanolic stock or as lipoprotein complexes) did not elicit a significant activation of these nuclear receptors. Moreover, vitamin E did not alter the responses of these receptors to their *bone fide* ligands when added together (Supplementary Information). These findings indicate that vitamin E's effects do not incur *via direct* binding to these receptors. Nevertheless, the possibility remains that nuclear receptor(s) are involved in an indirect manner (*i.e.*, through the action of intermediary metabolites) or that yet-unidentified transcriptional coregulators are required for this activity.

We believe that the concentrations of vitamin E used here (50–100 μM) are physiologically relevant for two reasons. First, these values are not far above those measured in plasma of healthy humans (10–30 μM; *e.g.*, (3, 85)). Second, we did not observe any competitor effects when vitamin E was added together with established ligands for these receptors (Supplementary Information).

In summary, our data show that in addition to its established function as a lipid-soluble antioxidant, α-tocopherol possesses transcriptional activities that are distinct and independent from the vitamin's role in regulating cellular redox. Future studies will focus on the molecular mechanisms and physiological consequences of this activity.

## Experimental procedures

### Cell lines

IHHs were cultured in Dulbecco's modified Eagle's medium supplemented with 10% vitamin E-deficient (<1 μM α-tocopherol by mass spectroscopy) fetal bovine serum (Hyclone Laboratories) as described (52). For RNA-Seq experiments, cells were seeded in triplicate 60 mm dishes and incubated for another 24 h with 100 μM *RRR*-α-tocopherol, 100 μM 6-HMTC, or equal volume of ethanol (<0.5% v/v). Human embryonic kidney 293 cells were cultured in MEM supplemented with 10% fetal bovine serum. All cells were cultured at 37°C in a humidified atmosphere containing 5% CO_2_.

### Reagents

RSL-3 was a generous gift of Brent Stockwell (Columbia University). We are also grateful for the generous gifts of molecular constructs for nuclear receptor reporter transactivation assays made by Hung-Yin Gao (CWRU), Paul Dawson (Emory University), and David Mangelsdorf (UTSW).

### Chemical synthesis and characterization of 6-HMTC

The chemical synthesis and characterization of 6-HMTC is described in the Supporting Information section.

### RNA isolation, library preparation, and RNA-Seq

Following treatment, total RNA was isolated from fresh lysates using the RNEasy Mini kit according to the manufacturer's protocol (Qiagen), followed by quality analysis on an Advanced Analytical Fragment Analyzer using the Standard Sense RNA kit. Total RNA input quantities were normalized prior to sequencing library generation using the TruSeq Stranded Total RNA Globin kit. Libraries were sequenced on an Illumina NextSeq 550 instrument using a midoutput run design with paired end sequencing and 75 cycles.

### *In vitro* assessments of radical-trapping antioxidant activity of α-tocopherol and 6-HMTC

#### Inhibition of coautoxidation of STY-BODIPY and egg phosphatidylcholine liposomes

STY-BODIPY (10 μM) and DTUN (0.2 μM) were added to phosphatidylcholine liposomes (as extruded 100 nm unilammelar liposomes; 1 mM in PBS). STY-BODIPY absorbance (*ϵ*_*565 nm*_ = 123,676 M^−1^ cm^−1^) was monitored over time before and after addition of α-tocopherol or 6-HMTC to 2 μM.

#### Inhibition of coautoxidation of styrene (4.3 M) and the 1-phenylbutadiene-conjugated BODIPY

PBD-BODIPY (10 μM) was initiated by azobis(isobutyronitrile) (6 mM in phenyl chloride) at 37 °C.

PBD-BODIPY absorbance (ε_591 nm_ = 139,000 M^−1^ cm^−1^) was monitored over time before and after addition of α-tocopherol or 6-HMTC to 8 μM.

### Induction and measurement of reactive oxygen species

IHH cells were treated with either 6-HMTC or α-tocopherol (in ethanolic stocks) for 16 h. Cells were challenged the next day with the GPX-4 inhibitor RSL-3 (3.5 μM) to induce lipid peroxidation for 1 h. Cells were then washed with Hanks' balanced salt solution and incubated with DCF diacetate in Hanks' balanced salt solution without phenol red (10 μg/ml) for 1 h before measuring DCF fluorescence using a plate reader (excitation = 485 nm; emission = 535 nm). DCF fluorescence was normalized to DNA content of the same well, determined by incubating the cells for 1 h with 2.5 μg/ml bisbenzimide in 2 M NaCl, 50 mM Na_2_HPO_4_, pH 7.4 in the dark at 37 °C. Bisbenzimide fluorescence was measured using a plate reader (excitation = 365 nm; emission = 460 nm).

### Bioinformatic analyses

The resulting demultiplexed FASTQ (86) paired-end reads were trimmed of adapters and filtered through the Skewer program. Reads with an average Phred quality score of less than 30 were filtered along with any reads with a length of less than 36 (87). The trimmed reads were then aligned to the National Center for Biotechnology Information reference genome for *Homo sapiens* using HISAT2 (National Center for Biotechnology Information, version GRCh38; (88)). The aligned reads were then counted and assigned to genes and associated metafeatures using the featureCounts program as a part of the Subread package (86). Libraries from the Bioconductor suite in the R programming language were used to analyze gene expression. A minimum count threshold of 1 count per sample was used to eliminate transcripts with a low median intensity below this threshold of detection. The edgeR Bioconductor package was used to perform trimmed mean of M-value normalization and log2 transformations (89). Distributions were assessed for normality and consistency between each sample, and no samples were removed because of inconsistent transcript distributions. Statistical analysis was performed and voom precision weights were applied to the dataset (90). The mean-variance trends were then assessed, and transcripts were filtered to eliminate duplicated counts and uncharacterized features.

### Nuclear receptor activation assays

To address the possibility that vitamin E regulates gene expression through direct activation of ligand-dependent transcription factors, we coexpressed in cultured cells the candidate nuclear receptors (*i.e.* PPARα/β/γ; liver X receptor, farnesoid X receptor, pregnane X receptor, or RAR-related orphan receptor) together with the appropriate reporter constructs in which expression of the luciferase gene is controlled by the specific response element(s). Details of these experiments, together with relevant data, are described in supporting information section.

## Data availability

All data presented and discussed here are contained within the article.

## Supporting information

This article contains supporting information (80, 81, 82, 83, 84, 85).

## Conflict of interest

The authors declare that they have no conflicts of interest with the contents of this article.
