# Supplementary material for: Antioxidant-independent activities of alpha-tocopherol
Source: J Biol Chem. 2025 Feb 18;301(4):108327. doi: 10.1016/j.jbc.2025.108327 (PMC11968272; doi:10.1016/j.jbc.2025.108327)
Supplement: Supplementary Information [file mmc1.pdf]

Supplementary Material 1: Organic Synthesis

NMR spectra were recorded in $CDCl_3$ (Cambridge Isotopes Laboratories) on either a Bruker Avance DPX-300 MHz or 400 MHz instruments. Bruker TOPSPIN 3.5 PL2 (400 MHz) and Bruker TOPSPIN 2.1 PL6 software (300 MHz) were used to analyse FID data. Chemical shifts were reported as δ values and coupling constants as *J*-values in Hertz (Hz). Trichlorofluoromethane ($CFCl_3$) was the internal standard for ^{19}F-NMR (282 MHz). Electron impact (EI) and fast atom bombardment (FAB) mass spectra were recorded on a Thermo Scientific high resolution double focusing magnetic sector mass spectrometer. Electrospray ionization (ES) used a Bruker HCT Plus Proteineer LC-MS.

Synthesis of (2*R*)-2,5,7,8-tetramethyl-2-(4,8,12-trimethyltridecyl)chroman-6-yl trifluoromethanesulfonate (**1**)

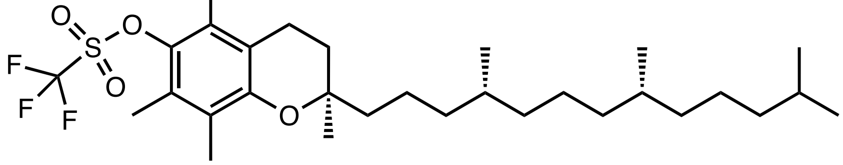

To solution of (+)-α-tocopherol (9.1 g, 21.1 mmol) and pyridine (4.8 mL, 59.3 mmol) in DCM (100 mL) was added trifluoromethanesulfonate anhydride (5.96 ml) at 0°C. After stirring for 1 h at room temperature, the reaction mixture was quenched with aqueous $NaHCO_3$ and extracted with DCM. The combined organic phase was dried over Na_2SO_4, filtered, and evaporated under reduced pressure to afford the title compound (11.2 g, 94%) as a clear oil. TLC: R_f = 0.62 (Hex/CH_2Cl_2, 5:1). ^{1}H-NMR (400MHz, $CDCl_3$) δ 2.62 (*t, J* = 6.80 Hz, 2H), 2.25 (*s*, 3H), 2.22 (*s*, 3H), 2.12 (*s*, 3H), δ 1.84 (*enant dt*, *J* = 6.80 Hz, 2H), 1.67 – 1.09 (several peaks, 21H) 0.88 (*m*, 12H). ^{13}C NMR (100 MHz, $CDCl_3$) 150.9, 139.7, 128.1, 126.7, 124.4, 118.5, 75.7, 40.0, 39.4, 37.3, 32.8, 32.7, 30.9, 28.0, 24.8, 24.4, 23.9, 22.7, 22.6, 21.0, 20.7, 19.8, 19.7, 19.6, 14.0, 13.2, 12.0. ^{19}F-NMR (282 MHz, $CDCl_3$) δ -73.59. HRMS (ESI) Calculated for $C_{30}H_{49}O_3F_3S$: 562.3304; found: 562.3300 ± 0.000183

Synthesis of (2R)-2,5,7,8-tetramethyl-2-(4,8,12-trimethyltridecyl)chroman , 3

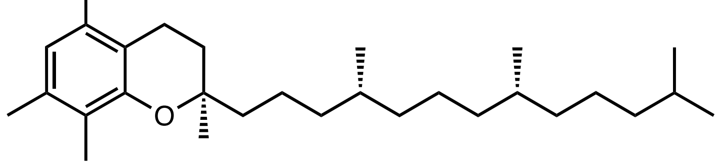

Compound **2** (11.18g, 19.87 mmol), and Fisher© brand palladium on carbon 10% (5.1g) were dissolved in THF (133 mL) before adding MeOH(67 mL) and triethylamine (12.2ml, 87.1 mmol) then shaken for 3 days under H_2 (35 psi) The reaction mixture was filtered through Celite, washed with EtOAc and the solvent evaporated. The residue was extracted with CH_2Cl_2 and H_2O. The water phase was washed with CH_2Cl_2 five times, the organic phases were combined, dried over Na_2SO_4 and evaporated to dryness. Silica gel column chromatography (Hex/CH_2Cl_2, 15:1) afforded **3** (8.12g, 98.6%) as a clear oil.

TLC: R_f = 0.45 (Hexane/CH_2Cl_2, 10:1) ^{1}H-NMR (400MHz, $CDCl_3$) 6.57 (*s*, 1H, Ar-H), δ 2.60 (*t, J* = 6.80, Hz, 2H, ArC*H*$_2$CH$_2$), δ 2.22 (*s*, 3H, ArCH$_3$), δ 2.18 (*s*, 3H, ArCH$_3$), δ 2.09 (*s*, 3H, ArCH$_3$), δ 1.82 (*enant dt*, *J* = 6.72 Hz, 2H, ArCH$_2$C*H*$_2$), δ 1.63 – 1.09 (*m*, 21H, phytyl-CH/CH$_2$ + 2'*R*-CH$_3$) δ 0.88 (*m*, 12H, phytyl-CH$_3$) ^{13}C-NMR (400MHz, $CDCl_3$) 151.56, 134.59, 133.32, 122.13, 122.00, 116.83, 75.09, 40.14, 40.06, 39.38, 37.56, 37.47, 37.30, 32.79, 32.70, 31.13, 31.07, 27.99, 24.82, 24.46, 24.01, 22.73, 22.64, 21.06, 20.11, 19.76, 19.70, 18.82, 11.35 MS [EI+] *m/z* 414 (M^+, 44%), 189 (13%), 149 (100%)
MS Calculated for $C_{29}H_{50}O$ 414.3862; found: 414.3857 ± 0.000082

Synthesis of (2R)-2,5,7,8-tetramethyl-2-(4,8,12-trimethyltridecyl)chroman-6-carbaldehyde (4)

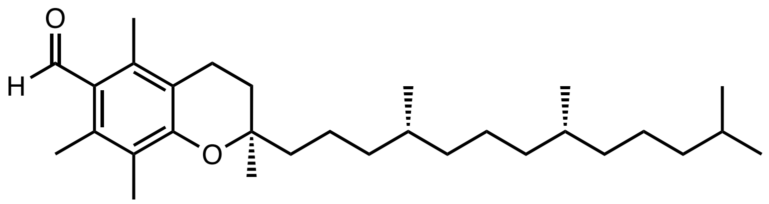

Titanium(IV)chloride 1M in toluene (48.4ml) was dissolved in dry DCM (57 mL) at 0°C under a nitrogen atmosphere. 3 (8.35g, 20.2 mmol) and α,α′-dichloromethoxymethane (3.56ml, 40.3 mmol) were dissolved in dry DCM (28 ml) under a nitrogen atmosphere then added dropwise over 15 minutes to the titanium(IV)chloride solution. The reaction was stirred for 1.5h, diluted with more CH_2Cl_2, then quenched slowly with water and stirred for 10 extra minutes. The phases were separated and the water phase was washed two times with CH_2Cl_2. The organic phases were combined and dried over Na_2SO_4 and evaporated down to dryness. Silica column chromatography (Hex/CH_2Cl_2, 1:1) afforded 3 (6.9g 77.3%) as a clear oil.
TLC: R_f = 0.55 (Hex/CH_2Cl_2, 1:1) ^{1}H-NMR (400MHz, $CDCl_3$) δ 10.58 (*s*, 1H, Ar-CHO), 2.67 (*t, J* = 6.80 Hz, 1H, Ar-C*H*$_2$CH$_2$), δ 2.50 (*s*, 3H, ArCH$_3$), δ 2.47 (*s*, 3H, ArCH$_3$), δ 2.15 (*s*, 3H, ArCH$_3$), δ 1.85 (*enant dt*, *J* = 6.80 Hz, 2H, ArCH$_2$C*H*$_2$), δ 1.68-1.04 (*m*, 21H, phytyl-CH/CH$_2$ + 2'*R*-CH$_3$) δ 8.88 (*m*, 12H, phytyl-CH$_3$) ^{13}C-NMR (400MHz, $CDCl_3$) 193.85, 155.74, 138.55, 138.21, 126.11, 123.53, 117.75, 77.23, 76.30, 40.04, 39.97, 39.38, 37.50, 37.45, 37.40, 37.36, 37.29, 32.80, 32.79, 32.67, 21.04, 30.99, 27.99, 24.82, 24.44, 23.95, 22.73, 22.64, 21.02, 20.34, 19.76, 19.69, 19.65, 19.59, 15.70, 14.88, 11.40 MS [EI+] *m/z* 442 (M+, 41%), 217 (22%), 177 (100%)
MS Calculated for $C_{30}H_{50}O_2$ 442.3811; found 442.3857 ± 0.000468

Synthesis of ((2R)-2,5,7,8-tetramethyl-2-(4,8,12-trimethyltridecyl)chroman-6-yl)methanol (6-HMTC)

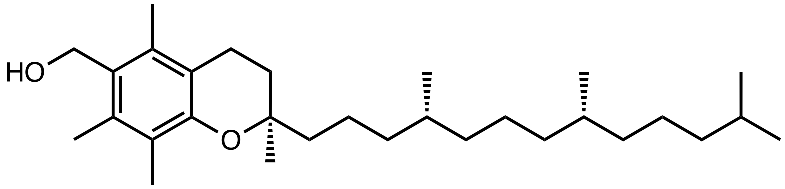

To a solution of **4** (6.9g, 15.6 mmol) in dry THF (50ml), lithium aluminum hydride (1.18g, 31.2 mmol) was added in two batches at 0°C under a nitrogen atmosphere. After 3.5h, the reaction mixture was diluted with DCM and quenched slowly with water and then brought to pH 6-7 with 1M HCl. (Note: Prolonged exposure to acidic conditions leads to dimerization of 6-HMTC.) The

phases were separated, and the water phase was washed 5x with CH_2Cl_2. The organic phases were combined and dried over Na_2SO_4 and dried down to dryness. Silica column chromatography (CH_2Cl_2 to CH_2Cl_2/MeOH 50:1) afforded 4 (6.9g, 97.7%) as a clear oil.
TLC: R_f = 0.2 (CH_2Cl_2) ^{1}H-NMR (400MHz, $CDCl_3$) δ 4.77 (*d*, *J* = 2.40 Hz 1H, Ar-C*H*$_2$OH), 2.65 (*t*, *J* = 6.80 Hz, 1H, Ar-C*H*$_2$CH$_2$), δ 2.35 (*s*, 3H, ArCH$_3$), δ 2.31 (*s*, 3H, ArCH$_3$), δ 2.15 (*s*, 3H, ArCH$_3$), δ 1.88 (*enant dt*, *J* = 6.80 Hz, 2H, ArCH$_2$C*H*$_2$), δ 1.66-1.05 (*m*, 21H, phytyl-CH/CH$_2$ + 2'*R*-CH$_3$) δ 8.88 (*m*, 12H, phytyl-CH$_3$) ^{13}C-NMR (400MHz, $CDCl_3$) 151.56, 134.62, 133.36, 127.90, 122.69, 117.26, 75.10, 59.82, 40.08, 40.02, 39.38, 37.57, 37.46, 37.30, 32.79, 32.72, 31.37, 31.32, 27.99, 24.83, 24.46, 23.91, 22.74, 22.64, 21.06, 20.93, 19.76, 19.70, 19.66, 19.60, 15.65, 14.72, 11.90 MS [EI+] *m/z* 444 (M+, 37%), 217 (17%), 177 (76%)
MS Calculated for $C_{30}H_{52}O_2$ 444.3967; found 444.3857 ± 0.000118

<u>Supplementary Material 2:</u> ***Nuclear receptor assays.*** To examine the possibility that vitamin E modulates gene expression through impacting hormone nuclear receptors, we employed specific reporter assays. In each case, we transfected cultured cells with expression vector for the nuclear receptor, alongside a reporter construct in which expression of the firefly luciferase is driven by the appropriate response element using Lipofectamine 2000 (Invitrogen, Carlsbad, CA). After 24 hours, an appropriate ligand (positive control, tocopherol, or solvent control) was added, and luminescence was measured using the Dual glow Luciferase Assay (Promega) in a Tecan Spark plate reader. Firefly luminescence was normalized to that of Renilla (co-transfected in the pRL-TK vector). Transfections were done in 4-9 replicates in 96-well plates using 120 ng of luciferase reporter construct, 30 ng of receptor expression vector, and 15 ng of the Renilla pRL-TK plasmid. In each case, α-tocopherol was added from an ethanolic stock, as well as a lipoprotein-bound complex prepared as described earlier (80). Reagent combinations for each nuclear receptor are provided in the Table below as well as representative data.

Table S2: nuclear receptor reagents.

**Nuclear receptor**	**Receptor/Reporter plasmid**	**positive control ligand**	**References**
PPARa	pCMX-mPPARa + pTK-Luc-3X-PPARE	fenofibrate	(81)
PPARb/d	pCMX-mPPARb + pTK-Luc-3X-PPARE	GW501516	(82)
PPARg	pCMX-mPPARg + pTK-Luc-3X-PPARE	rosiglitazone	(83)
FXR	pCMX-mFXR + pTK-Luc-FXRE	GW4064	(84)
LXR	pCMX-mLXR + pLXRE-Luc	GW3965	(85)
PXR	pSG-hPXR + pTK-LUC-PXRE;	SR12813	(84)

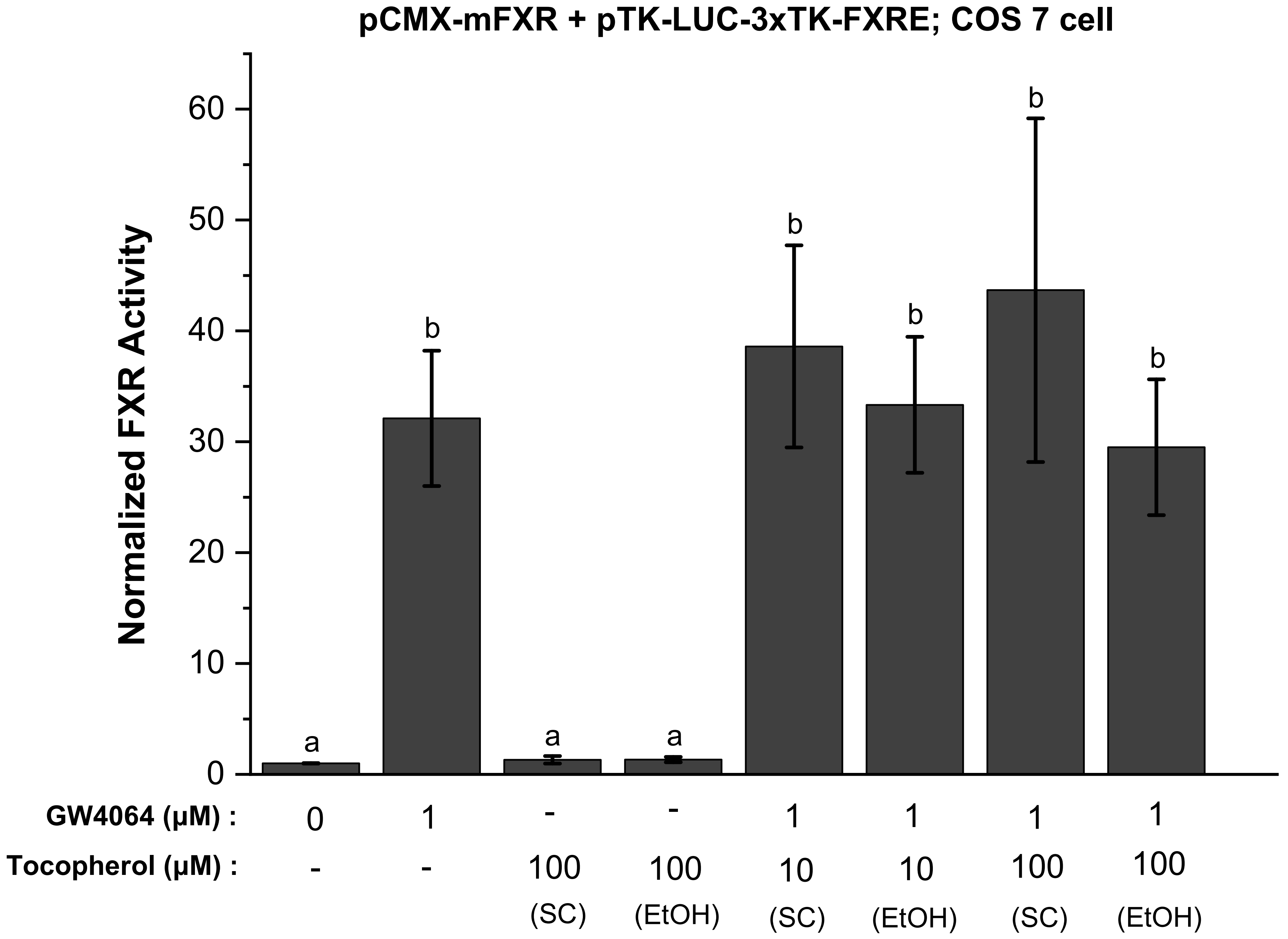

pCMX-mFXR + pTK-LUC-3xTK-FXRE; COS 7 cell
Normalized FXR Activity
0
10
20
30
40
50
60
a
b
a
a
b
b
b
b
GW4064 (μM) :
0
1
-
-
1
1
1
1
Tocopherol (μM) :
-
-
100
(SC)
100
(EtOH)
10
(SC)
10
(EtOH)
100
(SC)
100
(EtOH)

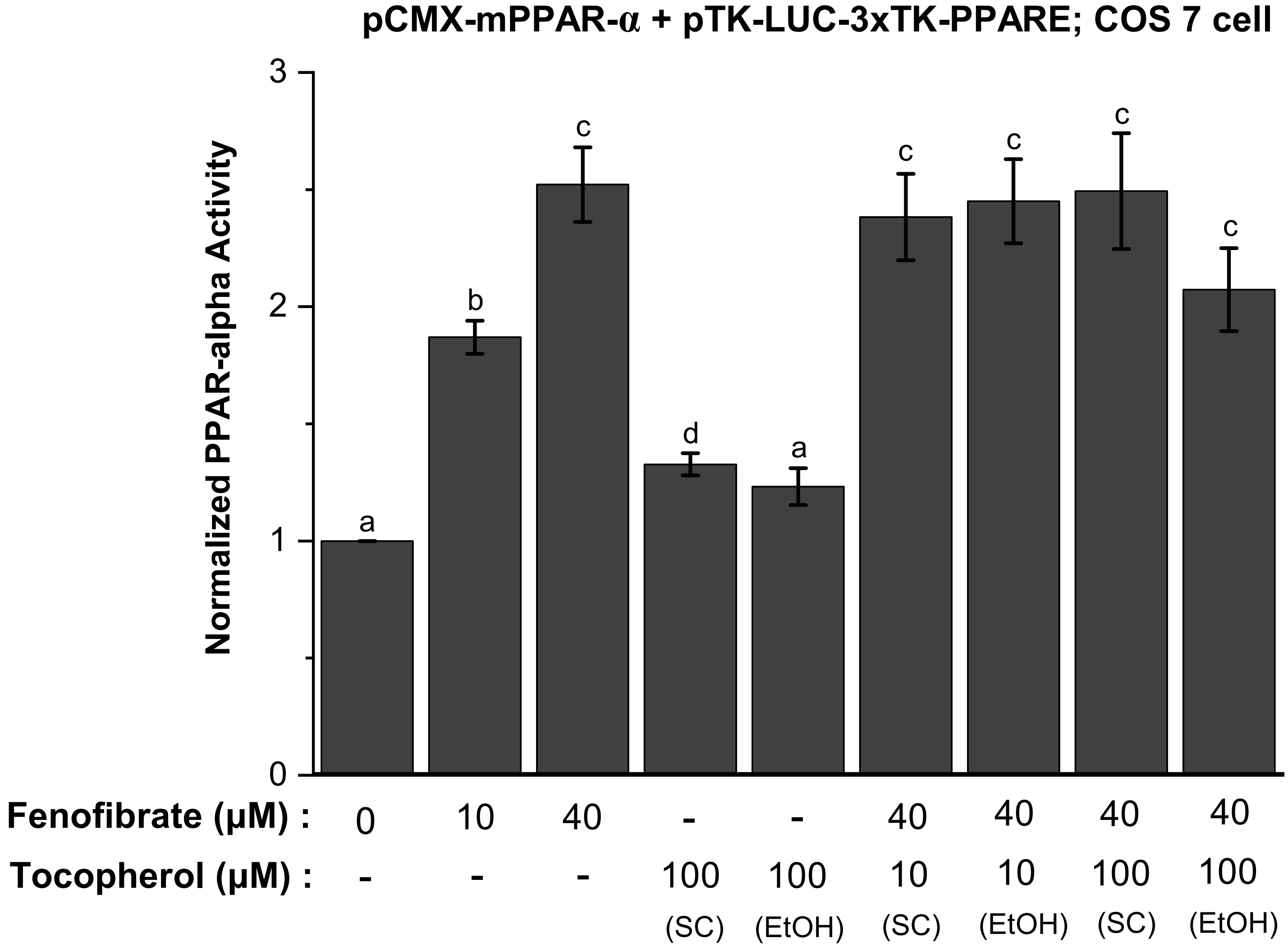
pCMX-mPPAR-α + pTK-LUC-3xTK-PPARE; COS 7 cell
Normalized PPAR-alpha Activity
0
1
2
3
a
b
c
d
a
c
c
c
c
Fenofibrate (μM) :
0
10
40
-
-
40
40
40
40
Tocopherol (μM) :
-
-
-
100
(SC)
100
(EtOH)
10
(SC)
10
(EtOH)
100
(SC)
100
(EtOH)

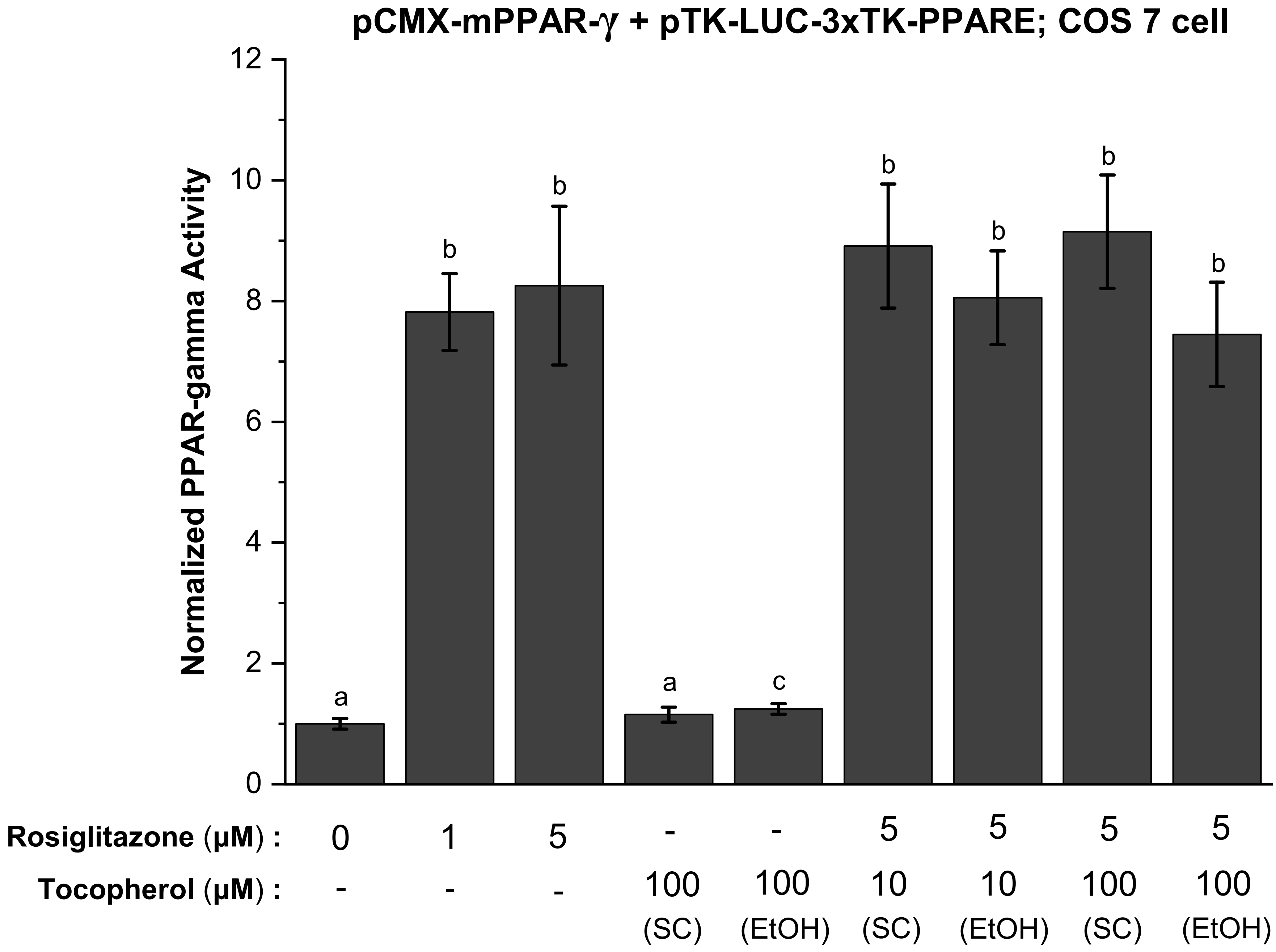
pCMX-mPPAR-γ + pTK-LUC-3xTK-PPARE; COS 7 cell
Normalized PPAR-gamma Activity
0
2
4
6
8
10
12
a
b
b
a
c
b
b
b
b
Rosiglitazone (μM) :
0
1
5
-
-
5
5
5
5
Tocopherol (μM) :
-
-
-
100 (SC)
100 (EtOH)
10 (SC)
10 (EtOH)
100 (SC)
100 (EtOH)

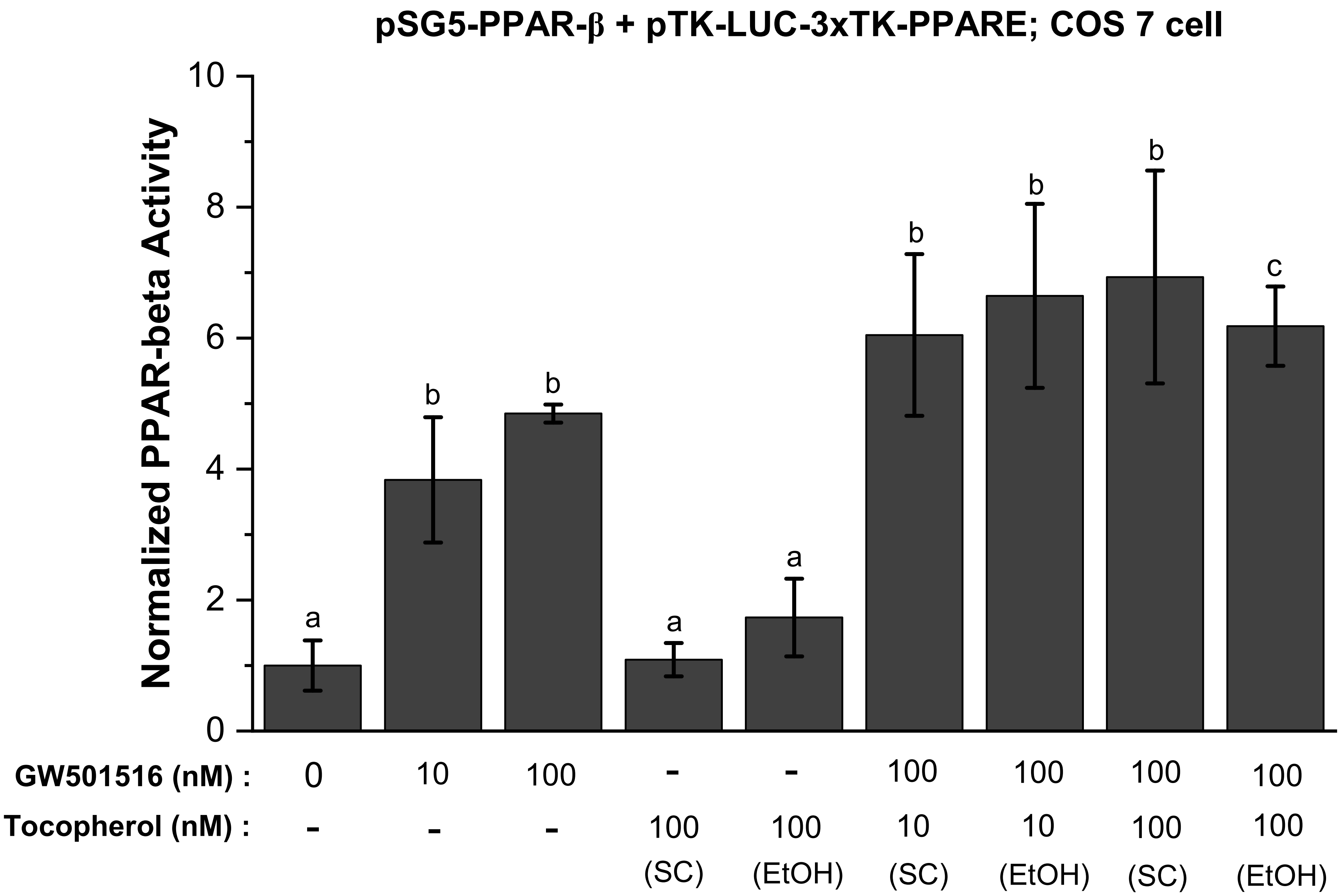
pSG5-PPAR-β + pTK-LUC-3xTK-PPARE; COS 7 cell
Normalized PPAR-beta Activity
0
2
4
6
8
10
a
b
b
a
a
b
b
b
c
GW501516 (nM) :
0
10
100
-
-
100
100
100
100
Tocopherol (nM) :
-
-
-
100 (SC)
100 (EtOH)
10 (SC)
10 (EtOH)
100 (SC)
100 (EtOH)

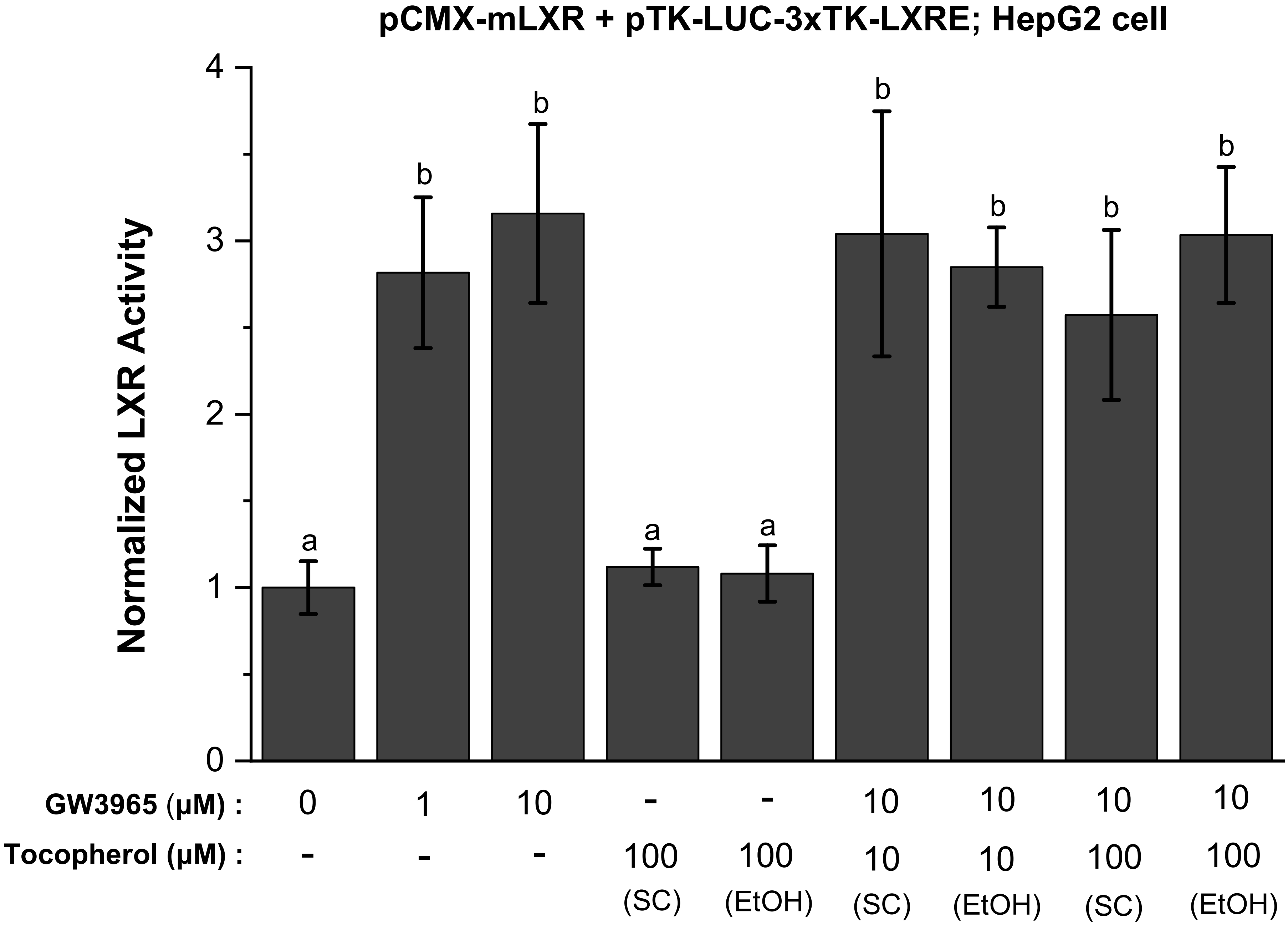
pCMX-mLXR + pTK-LUC-3xTK-LXRE; HepG2 cell
Normalized LXR Activity
0
1
2
3
4
a
b
b
a
a
b
b
b
b
GW3965 (μM) :
0
1
10
-
-
10
10
10
10
Tocopherol (μM) :
-
-
-
100 (SC)
100 (EtOH)
10 (SC)
10 (EtOH)
100 (SC)
100 (EtOH)

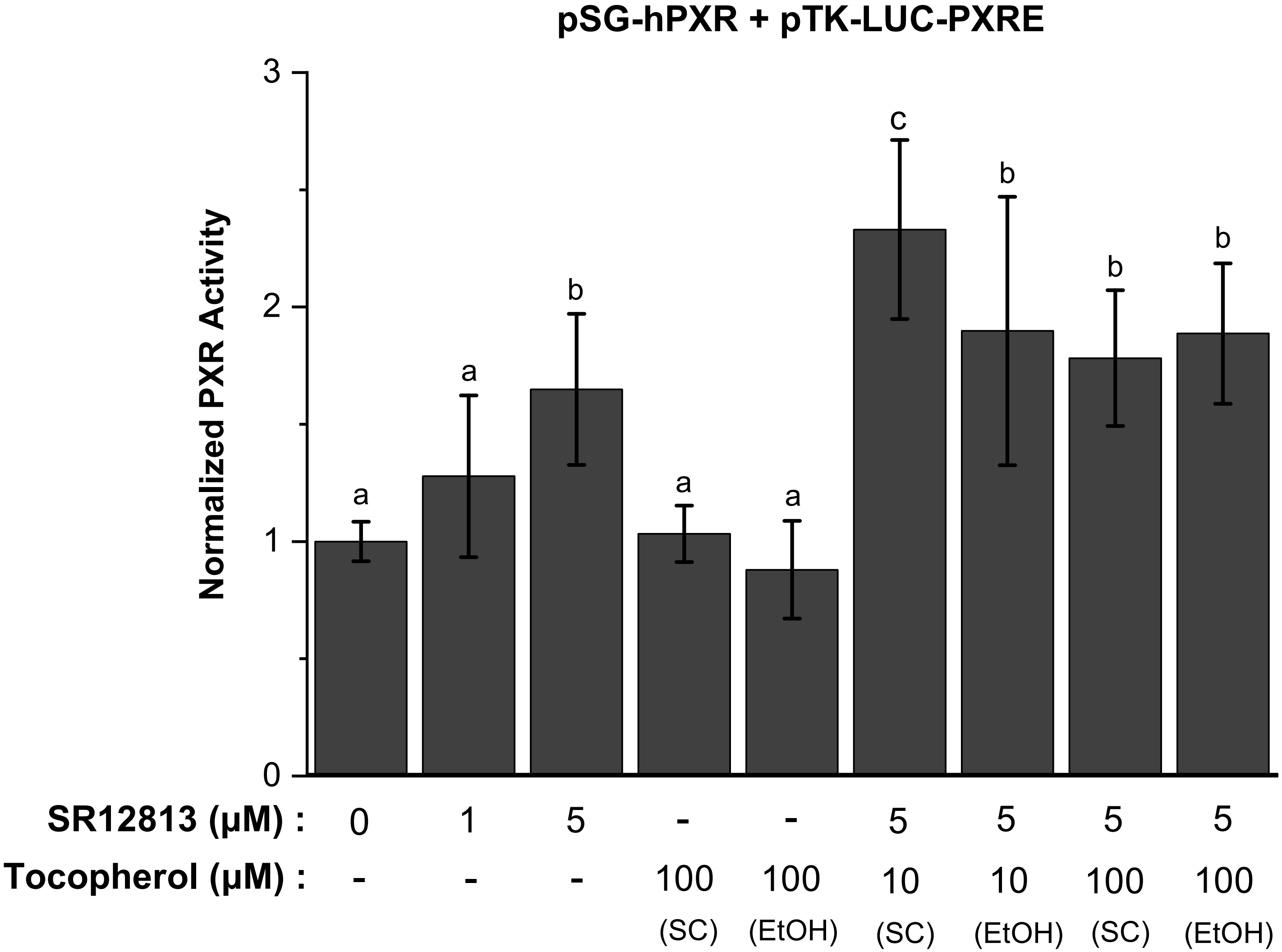
pSG-hPXR + pTK-LUC-PXRE
Normalized PXR Activity
0
1
2
3
a
a
b
a
a
c
b
b
b
SR12813 (μM) :
0
1
5
-
-
5
5
5
5
Tocopherol (μM) :
-
-
-
100
100
10
10
100
100
(SC)
(EtOH)
(SC)
(EtOH)
(SC)
(EtOH)
